# Optimization of Mechanical Properties and Evaluation of Fatigue Behavior of Selective Laser Sintered Polyamide-12 Components

**DOI:** 10.3390/polym16101366

**Published:** 2024-05-10

**Authors:** David Sommer, Henry Stockfleet, Ralf Hellmann

**Affiliations:** Applied Laser and Photonics Group, University of Applied Sciences, Würzburger Straße 45, 63743 Aschaffenburg, Germany

**Keywords:** selective laser sintering, tensile strength, fatigue behavior, fatigue strength

## Abstract

In this paper, a comprehensive study of the mechanical properties of selective laser sintered polyamide components is presented, for various different process parameters as well as environmental testing conditions. For the optimization of the static and dynamic mechanical load behavior, different process parameters, e.g., laser power, scan speed, and build temperature, were varied, defining an optimal parameter combination. First, the influence of the different process parameters was tested, leading to a constant energy density for different combinations. Due to similarities in mechanical load behavior, the energy density was identified as a decisive factor, mostly independent of the input parameters. Thus, secondly, the energy density was varied by the different parameters, exhibiting large differences for all levels of fatigue behavior. An optimal parameter combination of 18 W for the laser power and a scan speed of 2666 mm/s was determined, as a higher energy density led to the best results in static and dynamic testing. According to this, the variation in build temperature was investigated, leading to improvements in tensile strength and fatigue strength at higher build temperatures. Furthermore, different ambient temperatures during testing were evaluated, as the temperature-dependent behavior of polymers is of high importance for industrial applications. An increased ambient temperature as well as active cooling during testing was examined, having a significant impact on the high cycle fatigue regime and on the endurance limit.

## 1. Introduction

The additive manufacturing (AM) of polymers is increasingly gaining industrial attention, as prototyping and small batch production are being quickly realized. Selective laser sintering (SLS) is especially of interest, representing one of the AM approaches that has become an integral part of modern manufacturing technologies, allowing for reliable and fast part production [1,2].

With SLS, polyamides (polyamide 12) are most commonly processed, e.g., exhibiting high thermodynamic stability and isotropic properties, suitable for mechanical purposes [3,4]. Due to powder-based manufacturing, the typical anisotropy of other additive techniques, e.g., fused deposition modelling (FDM) [5,6], is minimized, improving the predictability of the resulting stability as well as the reproducibility [7,8].

The tensile strength and the fatigue behavior in the build direction define the characteristic threshold point for the practical use of SLS-fabricated technical parts under strain conditions. Pilipovic et al. [9] investigated the correlation of printing parameters and the resulting tensile strength under static conditions. They found that the higher the applied energy density, the more stable the test specimen, as the powder melting within a specimen increases. But, under real conditions, the dynamic mechanical stability with respect to long-term load is of particular importance, being often more informative than the static load behavior.

The fatigue behavior of SLS-printed parts was characterized by several studies, focusing on investigations of crack formation and propagation as well as prolongation independent of the part porosity [10] and analyses of topology optimized parts [11] or temperature [12]. Furthermore, van Hooreweder et al. [13] described the fatigue behavior of SLS-printed parts, identifying the different regimes of fatigue but omitting the variation in building parameters.

As an understanding of the correlation between process parameters and material properties is of upmost importance for the application of manufacturing technologies, a stable processing window has to be determined, knowing its impacts on the build components [14,15]. As shown by several studies, laser-based melting processes require an adequate energy density for the generation of fully dense parts that exceed the minimum threshold [16]. Moreover, a variation in process parameters can lead to quality anomalies, affecting, e.g., security-relevant components in terms of their mechanical stability [17].

Against this background, we report a comprehensive study of process parameters in regard to static and, most importantly, dynamic mechanical load behavior as well as temperature-dependent fatigue properties. Different process parameters, e.g., scan speed, laser power, energy density, and build temperature, were varied, investigating the tensile strength and the fatigue limit. In addition, the temperature-dependent load behavior of polymers was tested, evaluating the impact of different ambient temperatures.

## 2. Materials and Methods

### 2.1. Selective Laser Sintering

**Figure 1:**
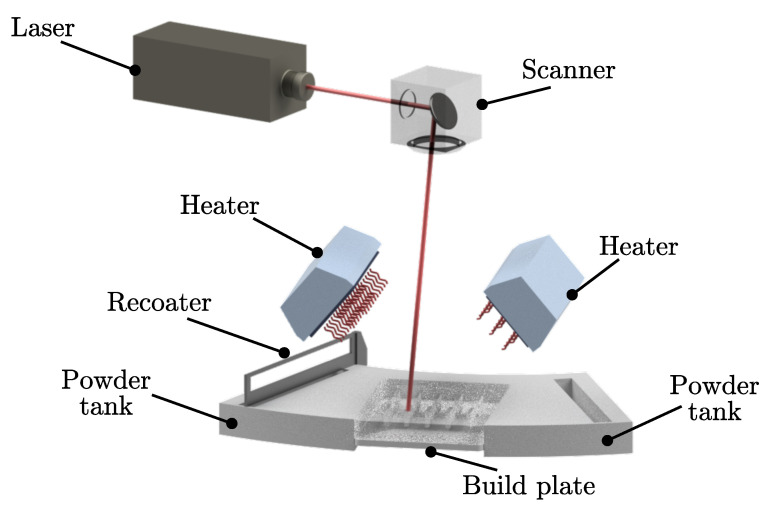
Schematic illustration of the selective laser melting unit.

For selective laser sintering, a Formiga P110 (EOS GmbH, Krailing, Germany) was employed, processing PA-12 polyamide standard material (PA2200). A schematic illustration of the relevant technical components in the build chamber is depicted in Figure 1. A CO_2_ laser with a maximum laser powder of *P* = 30 W was used, exhibiting a wavelength of λ = 10.6 µm and a nominal laser spot size of *d* = 500 µm at the focus position.

For optimization of the mechanical load behavior, different process parameters were varied, namely, the used laser power $P_{L}$ and the scan speed $v_{s}$, resulting in the applied energy density. As the layer height and the hatch distance were kept constant at $h_{l}$ = 100 µm, respectively, at $d_{h}$ = 0.25 mm, the applied areal energy density $\rho_{E}$ was calculated, following [18]:

(1) $$\rho_{E}=\frac{P_{L}}{v_{s}*d_{h}}$$

The used parameter combinations are shown in Table 1. To examine the effect of the variation in different process parameters, the impacts of laser power, scan speed, applied energy density, and build temperature were evaluated. Overall, an areal energy density of $\rho_{E}$ = 12–36 mJ/mm^2^ was considered in this study.

First, the laser power and the scan speed were varied, maintaining a constant energy density of $\rho_{E}$ = 30 mJ/mm^2^, as depicted in parameter sets 1–4 in Table 1. Varying scan speed and laser power resulted in dissimilar melt pool qualities, even though the same applied energy density was ensured. As the characteristics of fabricated components are determined significantly by the melt pool quality, a quantification of the static and dynamic mechanical load behavior was made.

Next, variations in the energy density were tested, as the laser power was decreased, keeping the scan speed constant. Starting at an applied energy density of $\rho_{E}$ = 36 mJ/mm^2^ ($P_{L}$ = 24 W), the laser power was reduced gradually to a used energy density of $\rho_{E}$ = 12 mJ/mm^2^ ($P_{L}$ = 8 W) (cf. Nos. 5–11). As the applied energy density directly affects the quality of fabricated components, an optimal parameter combination can lead to increased mechanical properties, improving tensile strength as well as fatigue behavior.

Finally, the impact of a varying the build temperature was tested. For this, the build temperature $\vartheta_{b}$ was varied in 4 steps from 168 °C to 174 °C, as shown by parameter sets 12–15 in Table 1. The applied energy density was kept constant at a semioptimal value of $\rho_{E}$ = 21 mJ/mm^2^ to avoid stagnant effects during testing.

### 2.2. Mechanical Testing

**Figure 2:**
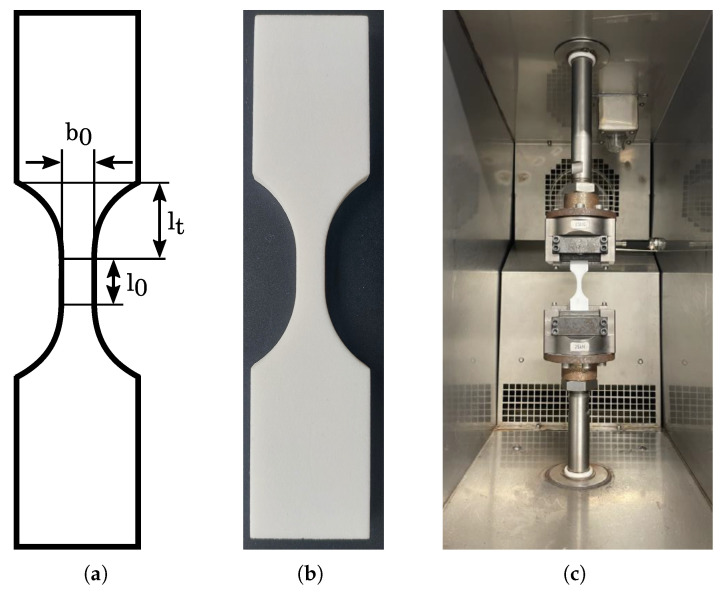
Standard tensile test specimen (**a**) with geometric dimensions (**b**) manufactured by selective laser sintering and (**c**) clamped in the gripping jaws in an electrodynamic actuator.

For mechanical testing, standard tensile specimens were manufactured, based on the DIN EN ISO 527 standard, exhibiting a thickness of $a_{0}\;=\;$6 mm and a width of $b_{0}\;=\;$6 mm, as shown in Figure 2a. Due to the dynamic testing and the limited distance between the clamping, the gauge length and the transition area were reduced to $l_{0}\;=\;$12 mm and $l_{t}\;=\;$17 mm, respectively. A selective laser sintered test component is shown in Figure 2b, which was manufactured with a very high geometric accuracy and without any superficial defects.

The static and dynamic mechanical testing was performed with an electrodynamic actuator UD020 (STEPLab, Resana, Italy). The tensile components were inserted centrally and clamped by a gripping jaw (ZwickRoell, Ulm, Germany), as shown in Figure 2c. For the static test, a tensile movement was performed, detecting the applied force as well as the performed strain, until final failure occurred. For the dynamic testing, an alternating load with a test frequency of *f* = 5 Hz was used, applying a symmetric tension compression load with a sinusoidal oscillation and a load ratio of R=−1, as shown in Table 2. The number of cycles, determined by a regulated load amplitude, was recorded and analyzed with a Wöhler diagram.

As temperature-dependent testing was performed, a climatic chamber was integrated, controlling the ambient temperature during testing. The tests were conducted at $\vartheta_{1}$ = 0 °C, $\vartheta_{2}$ = 20 °C, and $\vartheta_{3}$ = 40 °C, investigating the effects of active cooling as well as the influence of increased ambient temperature.

For the evaluation of the tensile strength, a batch of 5 specimens for every parameter set was tested, increasing the reliability of the results. For the UTS, an arithmetical mean as well as the standard deviation were calculated. In the graphical depiction, the trend for a single specimen is displayed, with the graphs superimposed on each other.

The investigation of fatigue performance was based on testing with 3 specimens for each amplitude, again calculating the arithmatic mean.

## 3. Results and Discussion

In this section, first, the results of the static and dynamic testing are presented. Specifically, the specimens subjected to different manufacturing parameters were compared using different scanning parameters, areal energy densities, as well as build temperatures. Secondly, the results of the temperatur-dependent testing are presented, revealing the impact of different ambient temperatures on the static and dynamic mechanical load behavior.

### 3.1. Variation in Manufacturing Parameters

**Figure 3:**
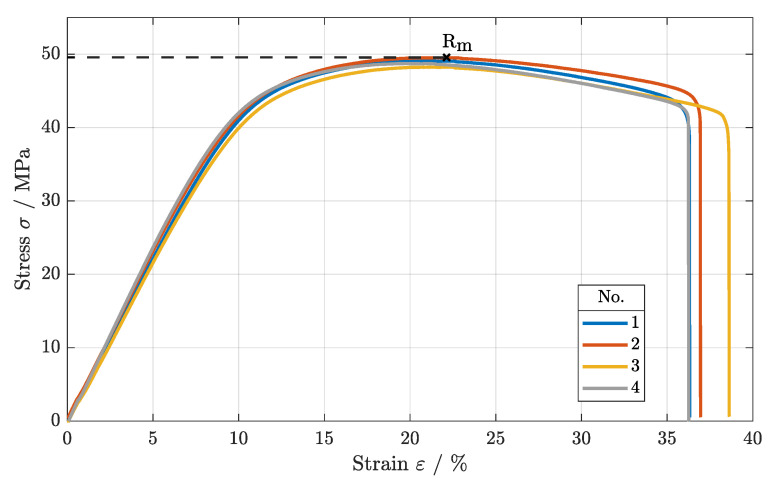
Stress–strain diagram of parameter sets 1–4, varying scan speed and laser power.

First, the tensile strength of the specimens of parameters sets 1–4 were investigated. The stress–strain diagram in Figure 3 shows that the tensile strength of all of the specimens varied between $R_{m}$ = 47.5 MPa and $R_{m}$ = 48.5 MPa (cf. Table 3), in accordance with [9,19,20]. Thus, variations in scan speed and laser power do not influence the tensile strength significantly, as long as the energy density is maintained, as shown in [9].

**Figure 4:**
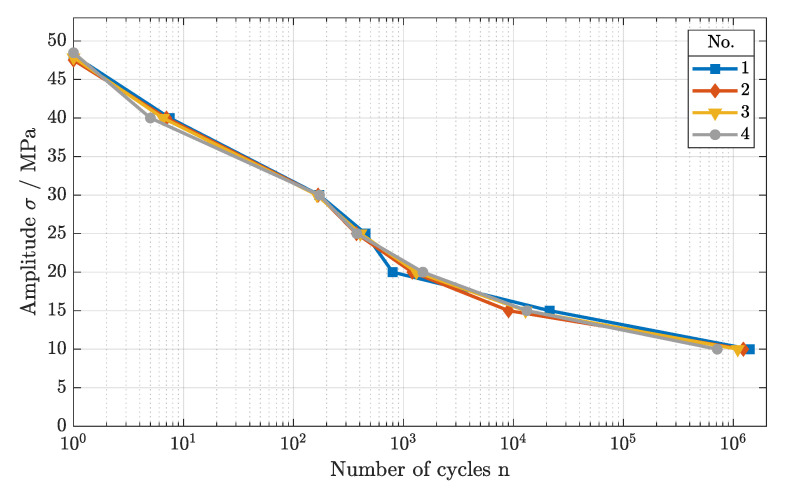
Fatigue behavior of components manufactured with 30 mJ/mm^2^, using different scan speeds and laser powers (cf. parameter sets 1–4).

For the dynamic testing, again, no significant difference was visible among the parameter sets, as shown in Figure 4. Starting at the maximum tensile strength of about $R_{m}$ = 48 MPa, the LCF regime was identified up to an applied load of 28 MPa for all parameter sets. Within the HCF, marginal differences were seen, as the slope of the fatigue curves were very similar, converging into the VHCF. Here, the endurance limit was identified at about 10 MPa, not exhibiting any failure during testing. Again, a possible process parameter interaction within the SLS process was excluded for the scan speed and the laser power. The static as well as the dynamic mechanical load behaviors did not differ as long as a constant energy density was maintained during the melting process.

**Figure 5:**
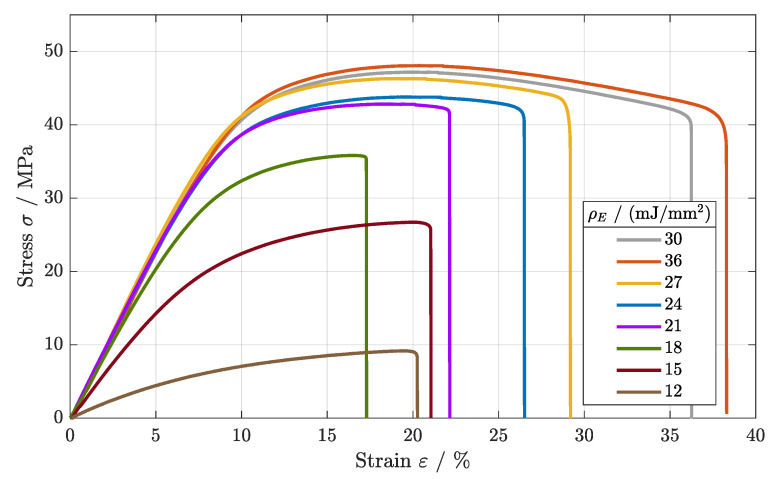
Stress–strain diagrams of parameter sets 4–11 for various applied energy densities.

The static mechanical load behavior of the specimens manufactured with different areal energy densities, starting with parameter set 4, is summarized in Figure 5. Beginning with an increase from $\rho_{E}$ = 30 mJ/mm^2^ to $\rho_{E}$ = 36 mJ/mm^2^, the tensile strength was not significantly improved in comparison to that of parameter set 4 ($\rho_{E}$ = 30 mJ/mm^2^). With $R_{m}$ = 48.5 MPa, this parameter set showed the maximum tensile strength within the parameter range, as shown in Table 4. A decrease in the energy density led to a reduction in the tensile strength to $R_{m}$ = 9.1 MPa ($\rho_{E}$ = 12 mJ/mm^2^). This could be attributed to the decreasing energy density as the melting quality of the specimens lowered, not further ensuring a sufficient fusion of powder particles [21]. Consequently, the tensile strength was affected negatively [9].

**Figure 6:**
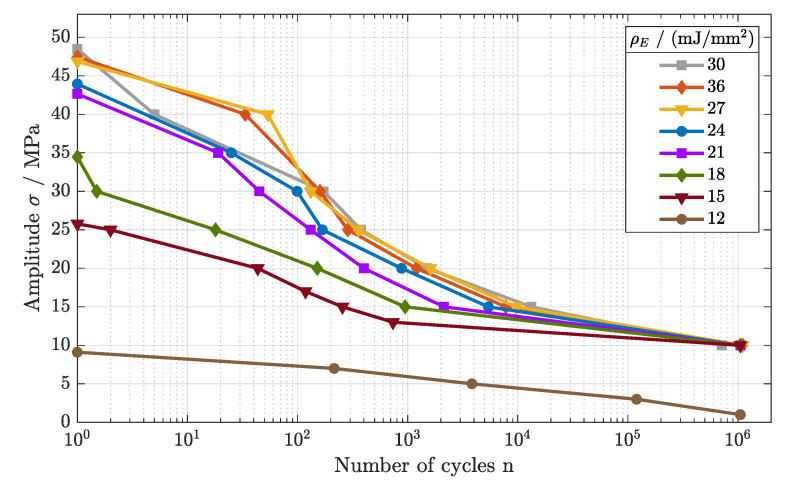
Fatigue behavior of components manufactured at different energy densities (cf. parameter sets 4–11).

In order to investigate the correlation between energy density and fatigue behavior, the Wöhler curves of parameter sets 4–11 were generated (cf. Figure 6). Similar to the tensile strength, the fatigue behavior differed significantly for different levels of energy density.

The Wöhler curves for the parameter sets 4–6 ($\rho_{E}$ = 27–36 mJ/mm^2^) show large overlaps for all regimes of the fatigue behavior. Starting at a tensile strength of $R_{m}$ = 47.5 MPa, the LCF regime was determined to an applied load of 40 MPa. After the turning point, the HCF developed between 18 MPa and 40 MPa with an analogous slope, merging into the VHCF regime.

For the energy densities of $\rho_{E}$ = 21– 24 mJ/mm^2^, again, a very similar trend was observed. Beginning with an offset of about 5 MPa in comparison to the parameter sets 4–6, the Wöhler curve shifted to lower amplitudes for the complete fatigue behavior, defining the LCF regime to 35 MPa and the HCF regime to 15 MPa. Within the HCF, the slope for the fatigue curve differed marginally, showing a steeper gradient for $\rho_{E}$ = 21 mJ/mm^2^, as the HCF started at lower cycle numbers. For the endurance limit, only a small offset was found.

A further lowering of the energy density ($\rho_{E}$ = 15–18 mJ/mm^2^) led to a pronounced reduction in the LCF and HCF regimes. Nonetheless, the VHCF, again, differed only marginally, defining an endurance limit of 10 MPa. Finally, a reduction in the energy to $\rho_{E}$ = 12 mJ/mm^2^ led to an inferior endurance limit, as no Wöhler curve was identifiable.

In conclusuion, a higher applied energy density led to increased tensile and fatigue properties. At higher applied energy densities, an elevated crystallinity was observed, and material discontinuity was reduced [2,18]. Better coherence and lower porosities within the material structure lead to improved fatigue behavior, as defects cause crack initiation and propagation [22]. For lower applied energy densities, porosities evolve due to a lack of fusion between two successive layers [23]. The results further led to the assumption of a temperature-dependent correlation, affecting the long-term stability of polyamide components.

As a higher applied energy density led to higher tensile strength as well as to improved fatigue behavior, in the following experiment, variations in the build temperature $\vartheta_{b}$ was tested. For the testing, a constant energy density of $\rho_{E}$ = 21 mJ/mm^2^ was used to avoid stagnant effects.

**Figure 7:**
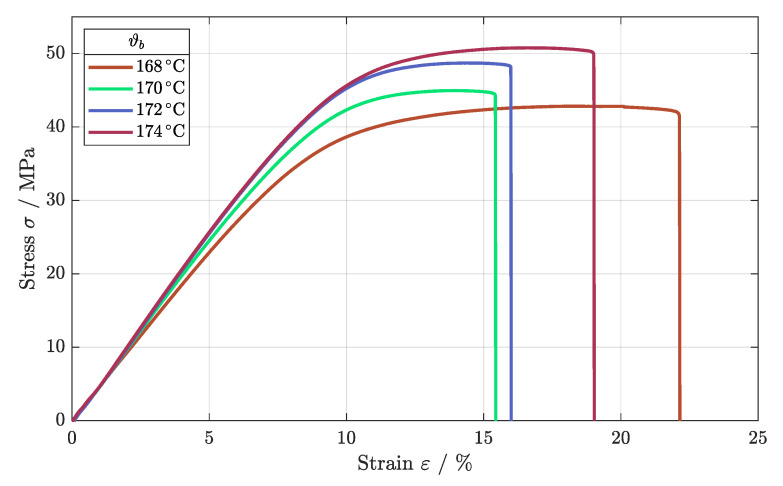
Stress–strain diagram for the different build temperatures.

The tensile strength of the SLS-built components increased with increasing build temperatures, as shown in Figure 7. Beginning at $R_{m}$ = 42.6 MPa, the tensile strength was increased up to $R_{m}$ = 50.2 MPa for a build temperature of $\vartheta_{b4}$ = 174 °C. Furthermore, the standard deviation reduced and the results had better reproducibility, as shown in Table 5. According to this, a higher build temperature can improve the melting quality of the structures, leading to the increased stability of the components.

**Figure 8:**
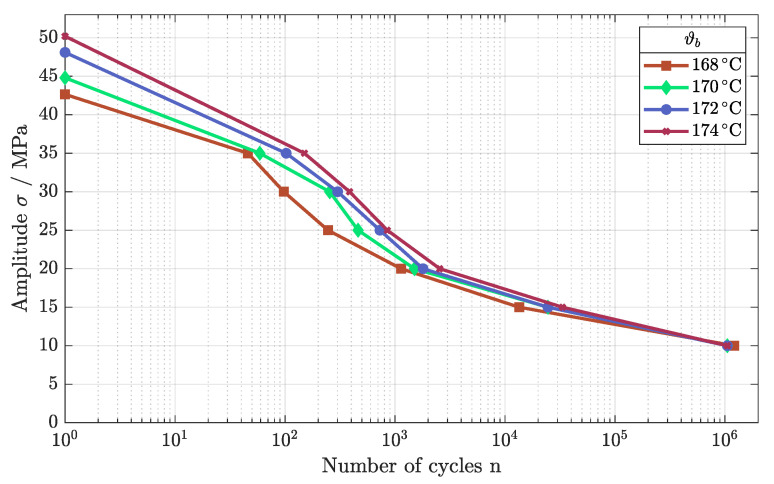
Fatigue behavior of components built as $\rho_{E}$ = 21 mJ/mm^2^ for different temperatures of the build chamber.

Next, the fatigue behavior was tested, evaluating the impact of higher build temperatures. Analogous to the tensile strength, the fatigue behavior strengthened as build temperature increased, as depicted in Figure 8. The different fatigue regimes were improved as the number of cycles differed significantly for the LCF as well as for the HCF regime. Only for the VHCF regime, the Wöhler curves merged, leading to an endurance limit of about 10 MPa for the different build temperatures. A reduction in build temperature and subsequently in the temperature of the powder bed led to a reduction in sintered density, affecting the fatigue behavior negatively [24].

### 3.2. Temperature-Dependent Testing

For the evaluation of the temperature-dependent mechanical load behavior, static and dynamic tests were performed at different ambient temperatures. Tests were performed at an elevated temperature of $\vartheta_{3}$ = 40 °C as well as with an active cooling at a temperature of $\vartheta_{1}$ = 0 °C, with the results compared to those obtained at room temperature. For this study, the optimized parameters were used, which were determined through the previously conducted experiments. An applied energy density of $\rho_{E}$ = 27 mJ/mm^2^ was maintained using a laser power of *P* = 18 W, a scan speed of $v_{s}$ = 2666 mm/s, and a hatch distance of $d_{h}$ = 0.25 mm. Additionally, a build temperature of $\vartheta_{b}$ = 174 °C was ensured during the build process.

**Figure 9:**
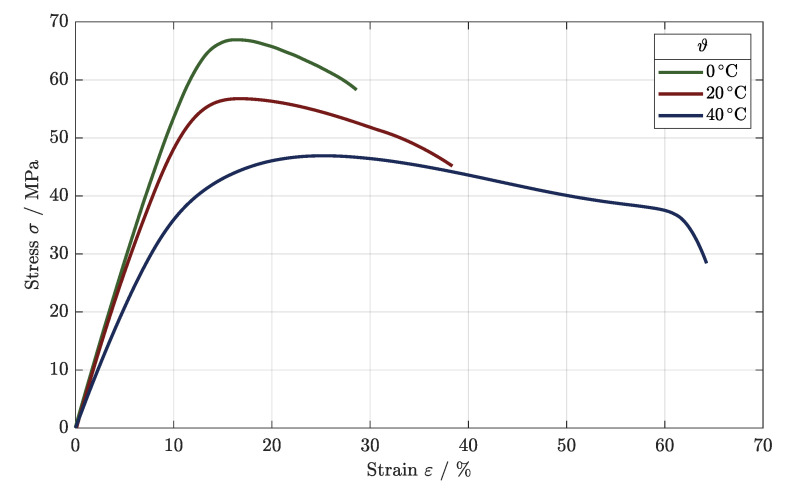
Stress–strain diagram for different tested ambient temperatures.

The stress–strain diagram in Figure 9 shows the typical load behavior of the tested polymers, as the tensile strength was reached, which was then followed by a long elongation after lateral contraction until the final failure occurred. Using the optimized parameter combination and the higher build temperature, a tensile strength of $R_{m}$ = 56.4 MPa was determined for the load at room temperature (cf. Table 6).

For the testing at $\vartheta_{1}$ = 0 °C, the development of the stress–strain correlation differed, as the area of the elongation was much smaller, and the maximum applied load increased to $R_{m}$ = 67.0 MPa. Due to the active cooling of the tensile component, a temperature-dependent elongation was prevented, as the tensile component’s temperature was maintained at about 0 °C. As such, the polymer structure was strengthened.

The tensile strength decreased at an elevated temperature of $\vartheta_{3}$ = 40 °C, as a rupture occurred at a maximum stress of $R_{m}$ = 47.0 MPa. Due to the higher temperature, the heating of the component as well as the elongation were encouraged, leading to a reduced charged area and a weaker tensile strength.

**Figure 10:**
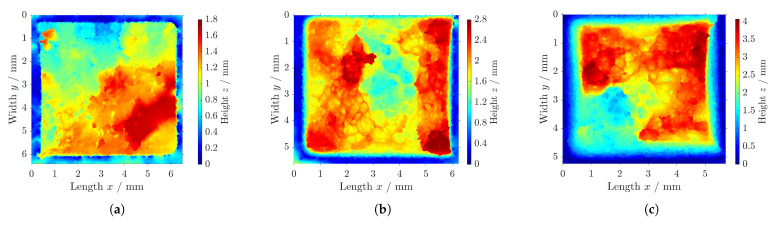
Fractographic analysis of fracture surfaces of specimens tested at (**a**) $\vartheta_{1}$ = 0 °C, (**b**) $\vartheta_{2}$ = 20 °C, and (**c**) $\vartheta_{3}$ = 40 °C.

As depicted in Figure 10, the fractographic analysis of the components at the tested temperatures shows different fracture behaviors, as the structure was affected during testing. Please note the differences in the scales for the height, as a single scale enables a better analysis of each fracture surface.

For the testing at $\vartheta_{2}$ = 20 °C, a distortion was visible, leading to a reduction in the test area and the final failure (cf. Figure 10b). A coarse-grained rupture occurred with a maximum difference in depth of Δz = 2 mm for the fracture surface.

As Figure 10a shows, the components in the testing at $\vartheta_{1}$ = 0 °C deformed slightly, as the test area only reduced marginally. The active cooling strengthened the structure, countering the load movement. Due to this, a very porous breakage with a plane fracture surface occurred, improving the mechanical load capacity. The fracture surface showed a maximum difference in height Δz = 1.2 mm.

In comparison to the testing at $\vartheta_{1}$ = 0 °C and $\vartheta_{2}$ = 20 °C, the testing at an elevated temperature showed very large elongation of the tensile components, as depicted in Figure 9. In the fractographic analysis, the deformation of the profile of the test component can be clearly seen (cf. Figure 10c). The test area reduced to below 5 mm × 5 mm as the component was elongated by the applied strain. The failure surface of the specimen showed large differences in height (Δz = 3.5 mm), as the structure ripped stepwise.

**Figure 11:**
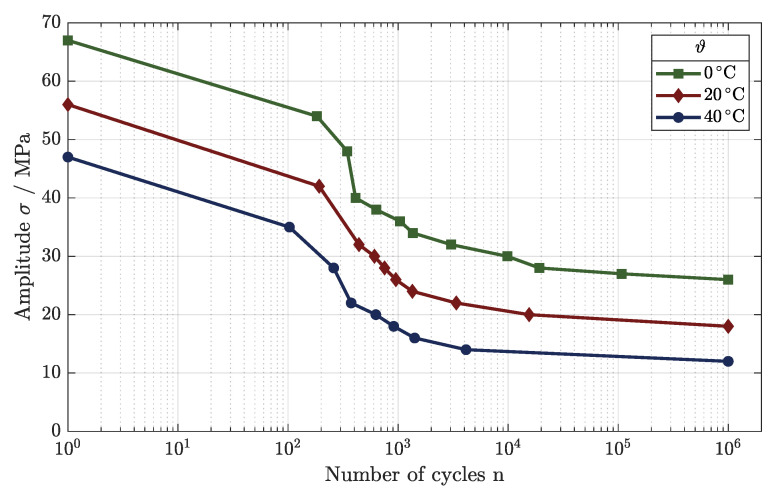
Temperature-dependent fatigue behavior of tensile components.

The fatigue behavior changed in very similar ways, as depicted in Figure 11, with different Wöhler curves for the tested ambient temperatures.

As observed for the variations in the parameters, for a load applied at room temperature, the LCF regime developed until an applied load of 32 MPa, leading to the HCF regime. This regime was detectable down to an applied load of 20 MPa, as the VHCF was reached at this point and the endurance limit could be set to 18 MPa.

For the testing at $\vartheta_{3}$ = 40 °C, the dynamic load behavior weakened in all areas of fatigue, lowering the applied loads significantly. Starting at $R_{m}$ = 47.0 MPa, the LCF could be identified down to a load of 22 MPa. After the turning point of the Wöhler curve, the HCF regime began until the VHCF regime was accomplished. The endurance limit was set to 12 MPa for operation at a temperature of $\vartheta_{3}$ = 40 °C.

Using active cooling during testing, the fatigue behavior improved significantly, as Figure 11 shows. The Wöhler curve shifted toward higher applied loads, and the HCF regime lengthened. The LCF regime developed for a maximum load of 40 MPa, showing an increase of 8 MPa in comparison to $\vartheta_{2}$ = 20 °C. As mentioned, the HCF improved, exceeding that achieved in the testing at room temperature by 2.5 times at σ = 30 MPa. The fatigue limit was set to 26 MPa, improving the endurance strength significantly by about 9 MPa.

## 4. Conclusions

In this study, the mechanical properties of polyamide-12 components were evaluated, investigating machining parameters as well as the impacts of different ambient temperatures.

The impact of different process parameters, specifically laser power, scan speed, and applied energy density, on the static and dynamic load behavior was studied via tests with manufactured standard tensile specimens. At first, different parameter combinations of scan speed and laser power were used, leading to a constant applied energy density as well as to very similar load behaviors. Thus, secondly, various energy densities were tested, resulting in significant differences in the static testing and fatigue behavior. For the optimal mechanical load behavior, a parameter combination of 18 W for the laser power and a scan speed of 2666 mm/s was found, exhibiting an applied energy density of 27 mJ/mm^2^.

The temperature-dependent mechanical properties were evaluated, with the tensile strength and the fatigue behavior was tested at different ambient temperatures. In this study, an decrease in tensile strength due to the elevated operation temperature were observed, and a significant improvement in fatigue behavior due to the active cooling of the components was achieved.

## Figures and Tables

**Table 1:** Process parameter sets for the manufacturing of tensile specimens.

No.	Laser Power	Scan Speed	Hatch Distance	Build Temperature	Energy Density
	**P/W**	**$v_{s}$/(mm/s)**	**$d_{h}$/mm**	**$\vartheta_{b}$/°C**	$\rho_{E}$ **/(mJ/${\mathrm{mm}}^{2}$)**
1	25	3333	0.25	168	30
2	22.5	3000	0.25	168	
3	15	2000	0.25	168	
4	7.5	1000	0.25	168	
5	24	2666	0.25	168	36
6	18	2666	0.25	168	27
7	16	2666	0.25	168	24
8	14	2666	0.25	168	21
9	12	2666	0.25	168	18
10	10	2666	0.25	168	15
11	8	2666	0.25	168	12
12	14	2666	0.25	168	21
13	14	2666	0.25	170	
14	14	2666	0.25	172	
15	14	2666	0.25	174	

**Table 2:** Instruments and parameters used for the mechanical testing.

Machine	STEPLab UD020
Test frequency *f*	5 Hz
Ambient temperature ϑ	0–40 °C
Amplitude σ	5–70 MPa
Load ratio *R*	−1
Gauge length $l_{0}$	12 mm
Area $S_{0}$	36 mm^2^

**Table 3:** Tensile strength of parameter sets 1–4, with constant applied energy density.

No.	$\rho_{E}$/(mJ/${\mathrm{mm}}^{2}$)	$R_{m}$/MPa
1	30	48.1 ± 1.04
2		47.5 ± 0.44
3		47.9 ± 0.45
4		48.5 ± 0.59

**Table 4:** Tensile strength of parameter sets 4–11 for various energy densities applied at a constant scan speed.

No.	$\rho_{E}$/(mJ/${\mathrm{mm}}^{2}$)	$R_{m}$/MPa
4	30	47.5 ± 0.59
5	36	48.5 ± 0.65
6	27	46.9 ± 0.77
7	24	43.9 ± 0.89
8	21	42.6 ± 1.28
9	18	34.5 ± 3.08
10	15	25.8 ± 1.08
11	12	9.1 ± 2.58

**Table 5:** Tensile strength for different build temperatures.

$\vartheta_{b}$/°C	$R_{m}$/MPa
168	42.6 ± 1.28
170	44.8 ± 0.48
172	48.1 ± 0.54
174	50.2 ± 0.49

**Table 6:** Tensile strength for different ambient temperatures.

ϑ/°C	$R_{m}$/MPa
0	67.0 ± 0.29
20	56.4 ± 0.46
40	47.0 ± 0.68

## Data Availability

All data are contained within this article.

## Abbreviations

The following abbreviations are used in this manuscript:

SLS	Selective laser sintering
FDM	Fused deposition modeling
LCF	Low cycle fatigue
HCF	High cycle fatigue
VHCF	Very high cycle fatigue
